# Crop cultivation without nitrogen fertiliser using nitrogen-fixing cyanobacterial extracts for low environmental impact

**DOI:** 10.1038/s41598-025-01741-5

**Published:** 2025-05-26

**Authors:** Yuji Haraguchi, Tatsuya Shimizu

**Affiliations:** Institute of Advanced Biomedical Engineering and Science, TWIns, Tokyo Women’s Medical University, 8-1 Kawada-cho, Shinjuku-ku, Tokyo, 162-8666 Japan

**Keywords:** Biotechnology, Environmental biotechnology, Microbiology, Environmental microbiology

## Abstract

In this study, we attempted to develop a biological fertiliser using the nitrogen-fixing cyanobacterium, *Trichormus* sp. PCC7120, with the aim of constructing a sustainable farming method for growing crops without chemical fertilisers. We attempted to hydroponically culture two types of rice (*Oryza sativa* L. ‘Sasanishiki’ and ‘Koshihikari’), broccoli (*Brassica oleracea* var. *italica*), and melon (*Cucumis melo* L.) using intracellular extracts from *Trichormus* sp. PCC7120, which was prepared using heat treatment or acid hydrolysis. Early crop growth, including length and weight, was compared among three groups: (i) pure water, (ii) chemical fertiliser, and (iii) *Trichormus* extract groups. Sasanishiki grew most efficiently in the 80% heat-treated and 2.5% acid-hydrolysed extracts than in pure water, whereas Koshihikari grew most efficiently in the 40% heat-treated extract than in pure water. Sasanishiki and Koshihikari consumed ammonium, potassium, and various proteinogenic amino acids in the *Trichormus* extract. Broccoli exhibited more length and weight when cultivated with 10–20% acid-hydrolysed extracts than in pure water and consumed glucose, along with phosphorus and glycine, in the extract. Melon exhibited more length and weight when cultivated with 10% acid-hydrolysed extracts than in pure water and actively consumed glucose, serine, glycine, and alanine in the extract. Crop growth with *Trichormus* extracts was comparable with that of the chemical fertiliser group. This report shows that ammonium, phosphorus, potassium, glucose, and amino acids in nitrogen-fixing cyanobacterial extracts contribute to crop growth, and these extracts may thus be valuable as biological fertilisers in crop cultivation.

**Supplementary Information:**

The online version contains supplementary material available at 10.1038/s41598-025-01741-5.

## Introduction

**Fig. 1:**
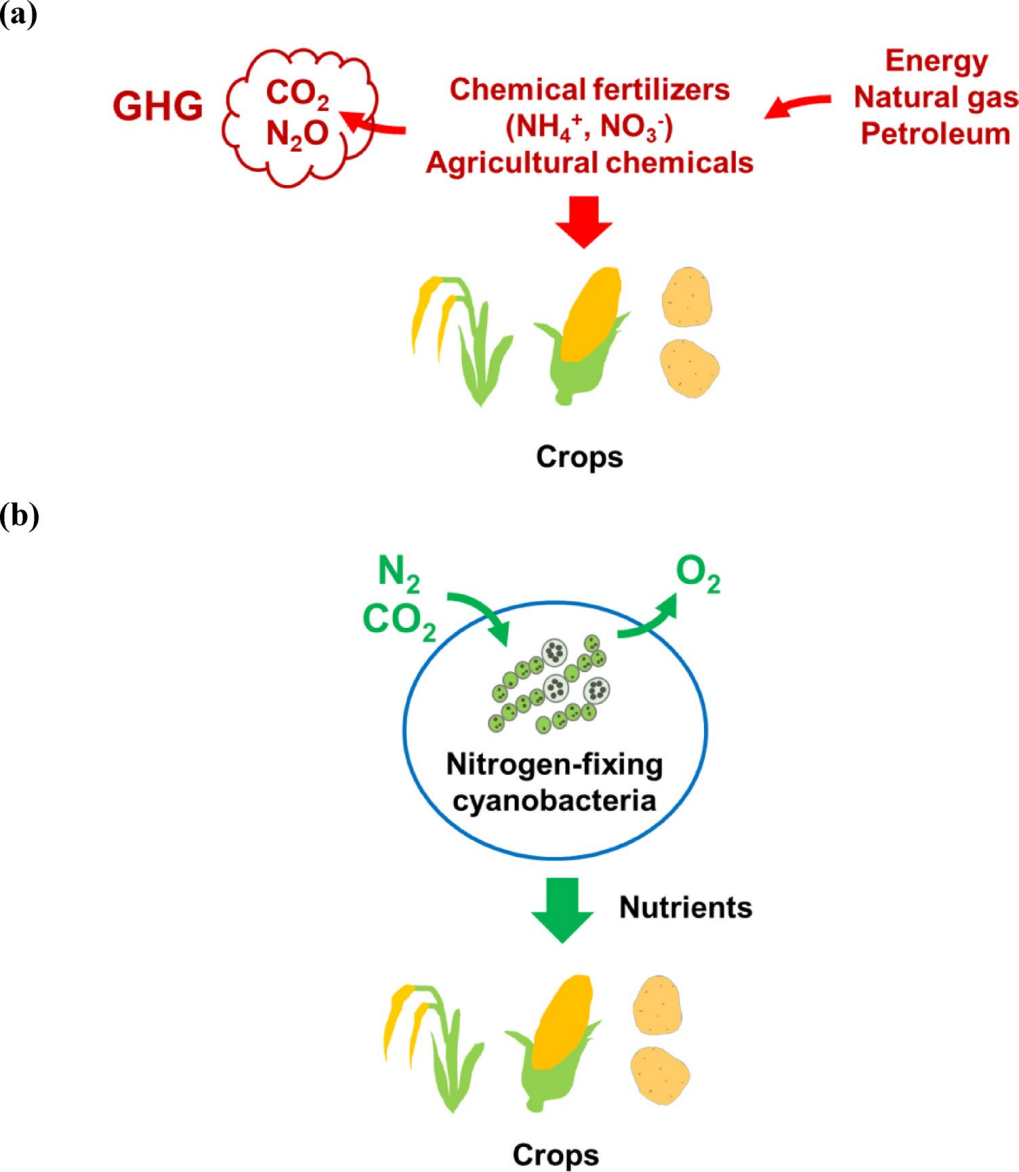
Schematic illustrating the issues with conventional crop cultivation using chemical fertilisers and agricultural chemicals, as well as the purpose of this study. (**a**) Conventional crop culture and food production. (**b**) Proposal for sustainable crop culture and food production. GHG: greenhouse gases.

The “Green Revolution” facilitated by modern agricultural techniques that use chemical fertilisers has greatly contributed to a stable food supply; the significant contributions to human welfare are too numerous to be comprehensively listed here. However, conventional agriculture consumes large amounts of energy and resources (Fig. 1a). Ammonia, the main fertiliser used for crop production, is synthesised via the Haber–Bosch process. This process is performed at high temperatures (300–500 °C) and pressures (200–300 atm) using pure hydrogen, resulting in the consumption of 1–2% of the annual energy supply, 3–5% natural gas, and 3% CO_2_ emissions^1–3^.

Some cyanobacteria fix CO_2_ and N_2_, produce nutrients (including glucose and amino acids), and release O_2_. Unlike chemical nitrogen fixation, which is carried out under extremely high temperatures and pressures, biological nitrogen fixation is carried out under normal temperatures and pressures and has a shallow environmental impact. In this study, to develop sustainable biological fertilisers, we attempted to culture rice (*Oryza sativa* L. ‘Sasanishiki’ and ‘Koshihikari’), which has a global production of approximately 757 million metric tons and the fourth highest production^4^, broccoli (*Brassica oleracea* var. *italica*), which produces 26 million metric tons (FAOSTAT. FAO, 2022), and melon (*Cucumis melo* L.), whose total production exceeds 30 million metric tons on a commercial scale in over 100 countries^5^, using extracts from the nitrogen-fixing cyanobacteria, *Trichormus* sp. PCC7120 (formerly named *Nostoc* and *Anabaena*) (Fig. 1b). This report demonstrates that nitrogen-fixing cyanobacterial extracts may be useful as biological fertilisers for crop cultivation and serves as the first step toward establishing a farming method for growing crops without the use of chemical fertilisers.

## Results

Explanations for each main figure are summarised in Supplementary Table 1.

### Sasanishiki culture using heat-treated *Trichormus* extracts

**Fig. 2:**
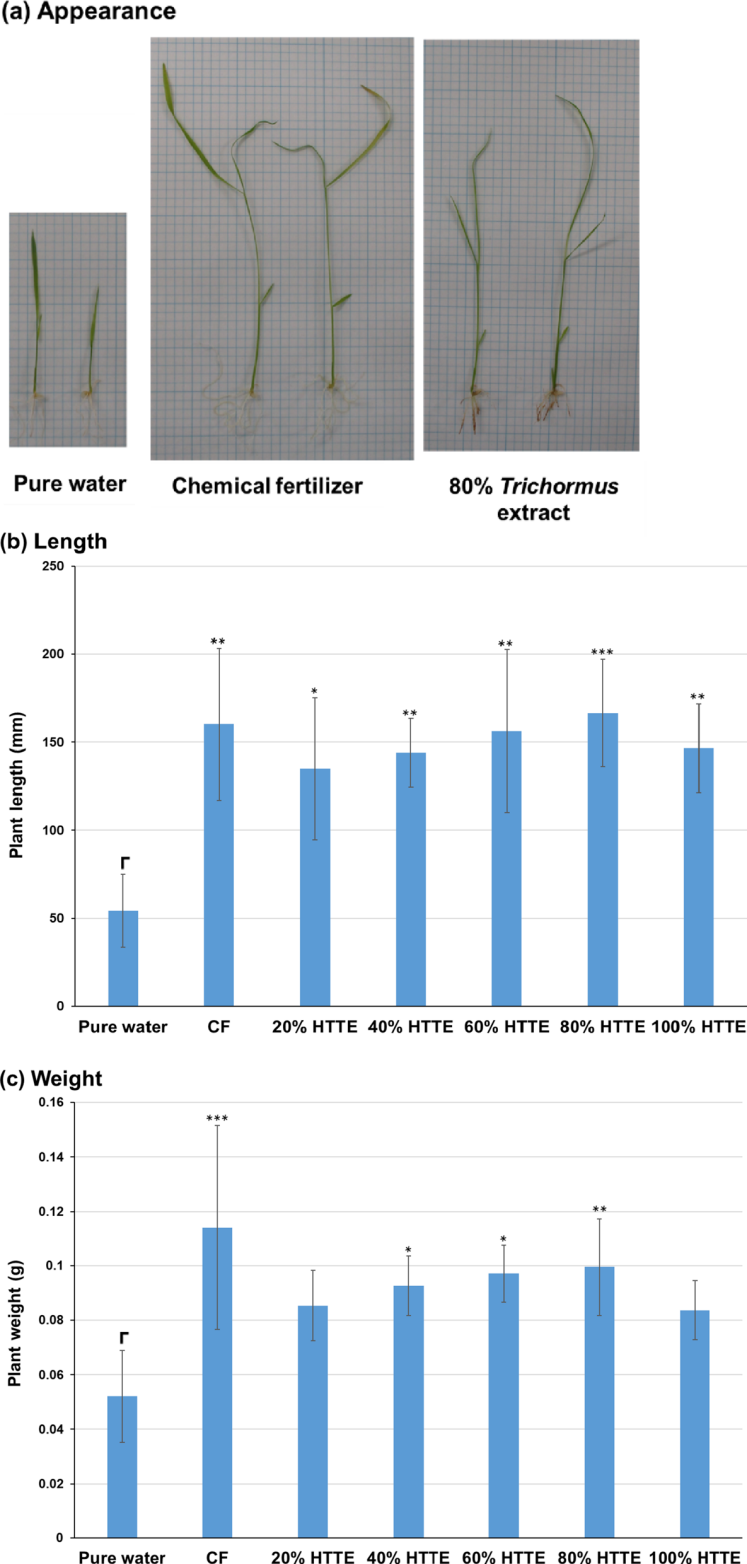
Sasanishiki cultivation in heat-treated *Trichormus* extract (20–100%). Appearance (**a**), length (**b**), and weight (**c**) of *Oryza sativa* L. ‘Sasanishiki’ assessed after 21-day cultivation. Data are presented as the mean ± standard deviation (*n* = 4). Statistical analyses were performed to compare pure-water cultivation with each cultivation condition tested. ^*^: *p* < 0.05; ^**^: *p* < 0.01; ^***^: *p* < 0.001; ^****^: *p* < 0.0001. CF: chemical fertiliser solution; HTTE: heat-treated *Trichormus* extract.

Initially, we attempted to cultivate Sasanishiki using a 2.5–40% heat-treated concentration of *Trichormus* extract. Sasanishiki grew well depending on the concentration of *Trichormus* extract, and the highest growth was observed at the 40% concentration (*n* = 3; data not shown). The growth of Sasanishiki showed 2.5 and 1.6 times greater length and weight compared to when it was grown in pure water. However, the effect of the extract on growth did not plateau even at 40%, prompting us to culture Sasanishiki in extract concentrations ranging from 20 to 100%. All concentrations of *Trichormus* extracts resulted in faster growth compared to using pure water, with Sasanishiki displaying the most efficient growth at the 80% extract (Fig. 2a–c). Compared to those grown in pure water, Sasanishiki showed 3.1 and 1.9 times greater length and weight, respectively, which was comparable to those grown with the chemical fertiliser solution. However, this growth-promoting effect was slightly diminished in the 100% extract.

### Sasanishiki culture using *Trichormus* extracts (acid hydration treatment)

We attempted to cultivate Sasanishiki using another *Trichormus* extract method: the acid hydration method (2.5–20% of the extracts). Sasanishiki growth was highest with the 2.5% extract, and as the extract concentration increased, growth tended to slightly decrease (Supplementary Fig. 2a–c). Sasanishiki growth when cultured in the 2.5% extract showed 2.1 and 1.5 times more length and weight, respectively, compared with those when grown in pure water, which was comparable with those when grown with the chemical fertiliser solution (Supplementary Fig. 2a–c). Efficient growth of Sasanishiki was observed with both heat-treated and acid-hydrolysed extracts. However, the heat-treatment method appeared to have a better growth effect.

### Metabolic analysis in Sasanishiki cultivation using heat-treated and acid-hydrolysed *Trichormus* extracts

**Fig. 3:**
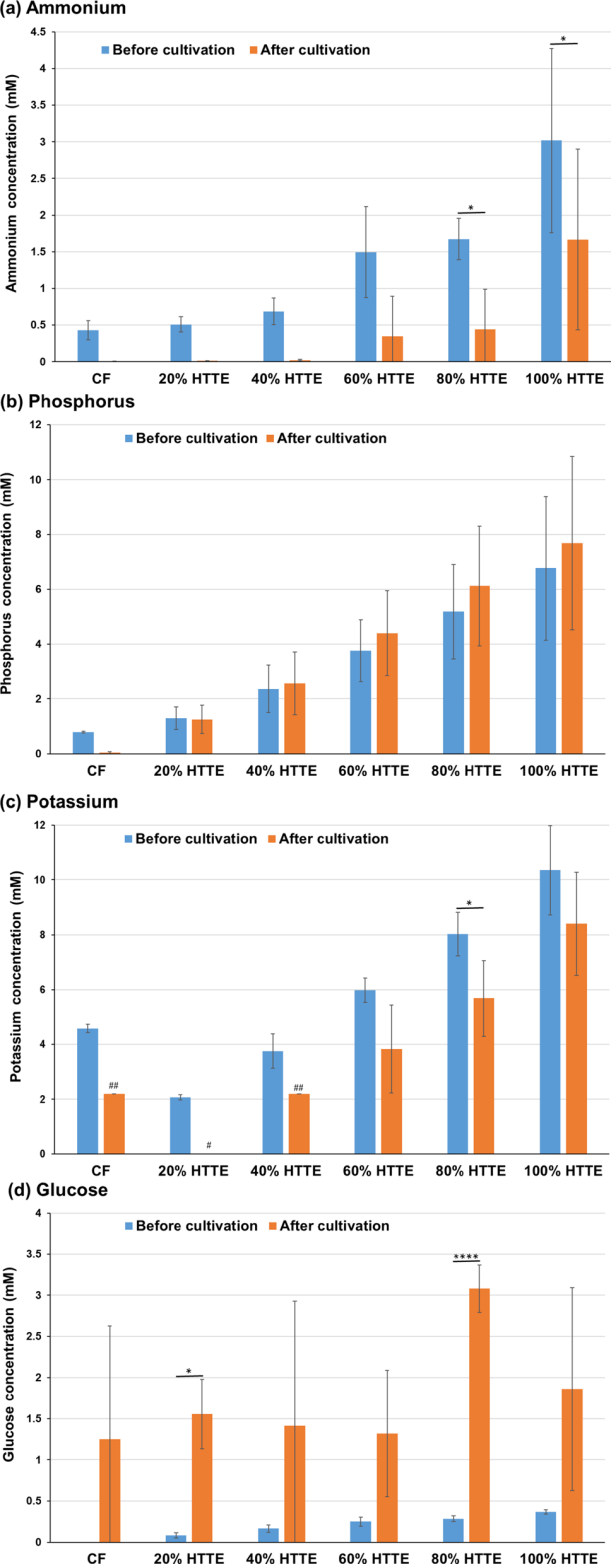

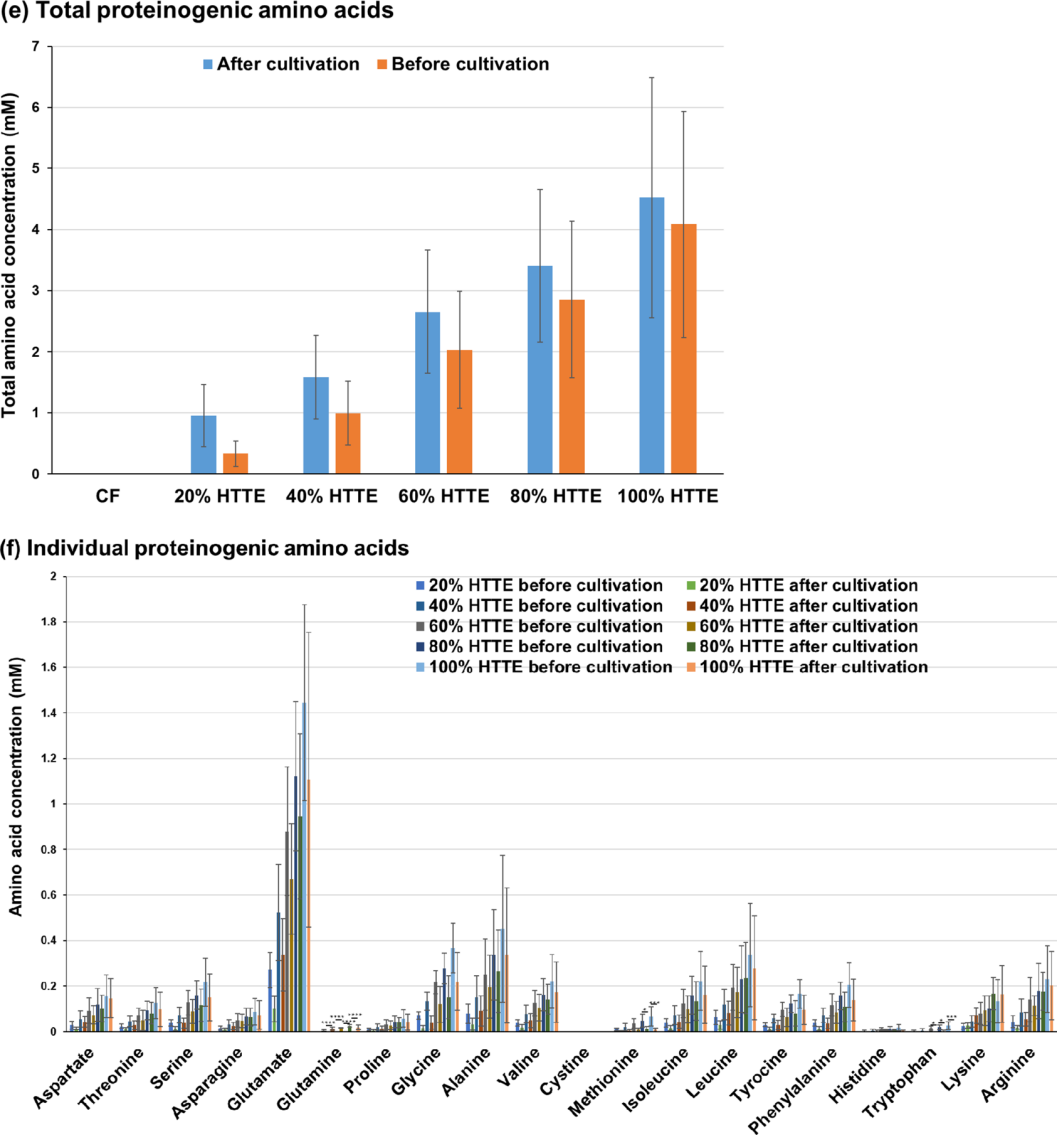
Sasanishiki metabolites of ammonium (**a**), phosphorus (**b**), potassium (**c**), glucose (**d**), and proteinogenic amino acids (**e**: total; **f**: individual) measured during cultivation (heat-treated Trichormus extract). Data are presented as the mean ± standard deviation (*n* = 4). Statistical analyses were performed to compare before and after cultivations. ^*^: *p* < 0.05; ^**^: *p* < 0.01; ^***^: *p* < 0.001; ^****^: *p* < 0.0001. ^#^ in the potassium data: Four of four trials were undetectable (< 1.5 mM). ^##^: Three of four trials were undetectable (< 1.5 mM), and the data in the graph show the one value that was detected. CF: chemical fertiliser solution; HTTE: heat-treated *Trichormus* extract.

Using the heat-treated *Trichormus* extract, we investigated the metabolism of Sasanishiki during cultivation. The amount of ammonium and phosphorus in the *Trichormus* extract was higher than that in the chemical fertiliser solution when more than 20% of the extract was added. Sasanishiki efficiently consumed ammonium in the extract and chemical fertiliser solution (Fig. 3a); however, it tended to consume phosphorus only in the chemical fertiliser solution (Fig. 3b). The amount of potassium in the extract was higher than that in the chemical fertiliser solution when more than 60% of the extract was added. Sasanishiki efficiently consumed potassium in the extract and chemical fertiliser solution (Fig. 3c). A trace amount of glucose was present in the extract but not in the chemical fertiliser solution (0.37 ± 0.03 mM in the 100% *Trichormus* extract; Fig. 3d). Sasanishiki did not consume glucose but rather excreted it (Fig. 3d). Proteinogenic amino acids were present in the extract but not in the chemical fertiliser solution (Fig. 3e). Sasanishiki tended to consume proteinogenic amino acids, including serine, glutamate, glycine, alanine, methionine, and tryptophan (Fig. 3e and f). However, Sasanishiki excreted a small amount of glutamine. At concentrations of 40% or more, the osmolality of the extract exceeded that of the chemical fertiliser solution (Supplementary Fig. 1a).

**Fig. 4:**
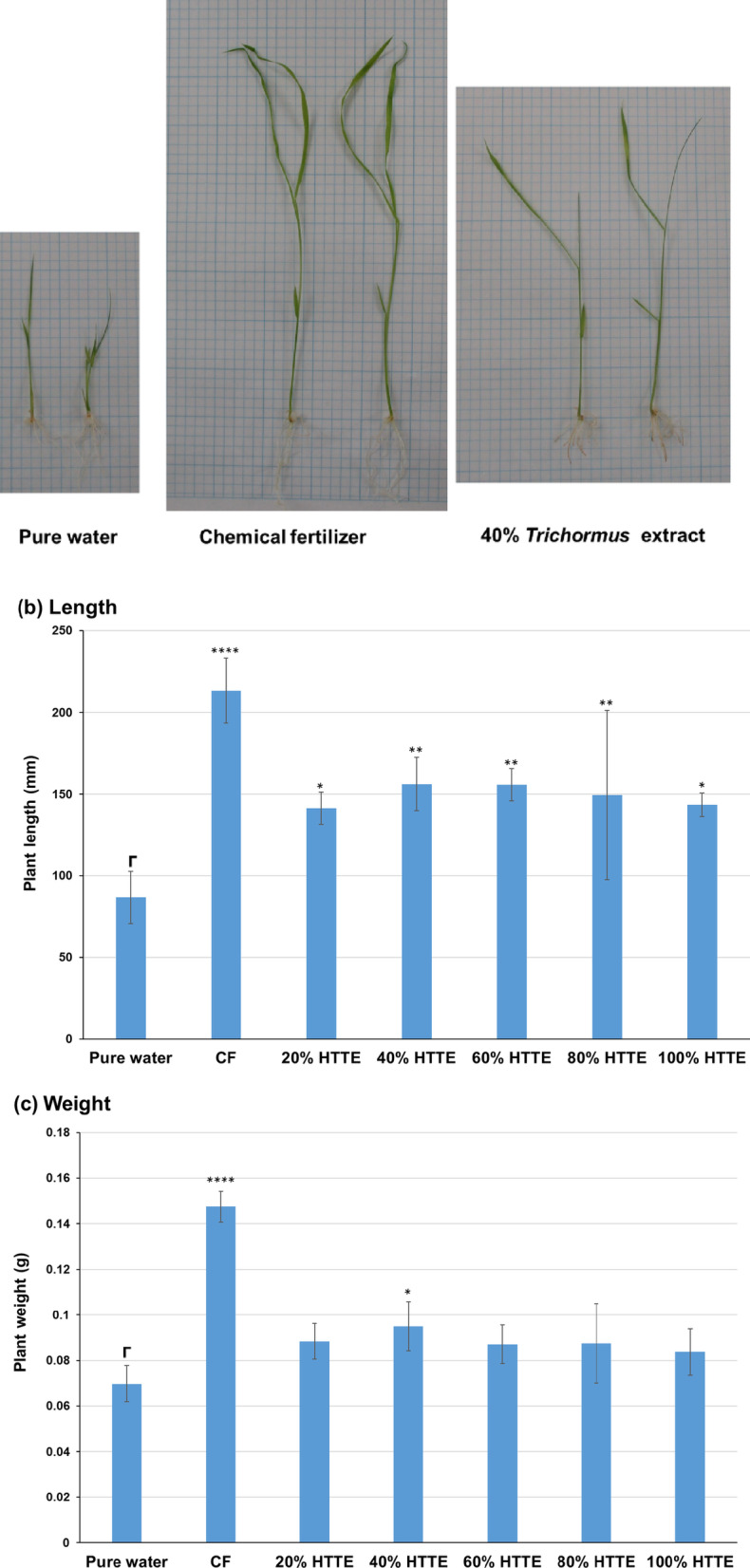
Koshihikari cultivation in *Trichormus* extract (20–100%). Appearance (**a**), length (**b**), and weight (**c**) of *Oryza sativa* L. ‘Koshihikari’ assessed after 21-day-cultivation. Data are presented as the mean ± standard deviation (*n* = 4). Statistical analyses were performed to compare pure-water cultivation with each cultivation condition tested. ^*^: *p* < 0.05; ^**^: *p* < 0.01; ^***^: *p* < 0.001; ^****^: *p* < 0.0001. CF: chemical fertiliser solution; HTTE: heat-treated *Trichormus* extract.

Next, the metabolism of Sasanishiki was analysed using an acid-hydrolysed *Trichormus* extract. The factors present in the heat-treated and acid-hydrolysed extracts included ammonium (100% heat-treated extract: 3.0 ± 1.3 mM; 20% acid-hydrolysed extract: 1.7 ± 0.2 mM), phosphorus (100% heat-treated extract: 6.8 ± 2.6 mM; 20% acid-hydrolysed extract: 1.9 ± 0.5 mM), potassium (100% heat-treated extract: 10.4 ± 1.6 mM; 20% acid-hydrolysed extract: 2.2 ± 0.1 mM), glucose (100% heat-treated extract: 0.37 ± 0.03 mM; 20% acid-hydrolysed extract: 5.6 ± 0.4 mM), and total amino acids (100% heat-treated extract: 4.5 ± 2.0 mM; 20% acid-hydrolysed extract: 7.2 ± 0.5 mM) (Fig. 4 and Supplementary Fig. 3). Notably, the 100% heat-treated extract had high concentrations of ammonium, phosphorus, and potassium, while the 20% acid-hydrolysed extract had high concentrations of glucose and amino acids. Sasanishiki actively consumed ammonia in the acid-hydrolysed extract (Supplementary Fig. 3a) and phosphorus in the low-concentration extracts (20% or less) (Supplementary Fig. 3b). Potassium levels were undetectable (< 1.5 mM) in extracts with concentrations below 20%; however, potassium consumption was observed in the 20% extract (Supplementary Fig. 3c). Sasanishiki did not consume glucose (Supplementary Fig. 3d). Sasanishiki consumed aspartate, serine, glutamate, glycine, and alanine, and the concentration of total amino acids decreased after cultivation (Supplementary Fig. 3e and f). However, Sasanishiki excreted a small amount of glutamine and lysine. The osmolality of the 2.5% extract was higher than that of the chemical fertiliser solution, and the osmolality of the 20% extract was more than 10 times that of the chemical fertiliser solution (Supplementary Fig. 1b).

### Koshihikari culture using *Trichormus* extracts and metabolic analysis

Next, we cultivated another rice cultivar, Koshihikari, using the heat-treated *Trichormus* extract (20–100%). All concentrations of the *Trichormus* extract caused faster growth than pure water, and Koshihikari growth with the 40% extract showed 1.8 and 1.4 times more length and weight, respectively, than those when grown in pure water (Fig. 4a–c).

**Fig. 5:**
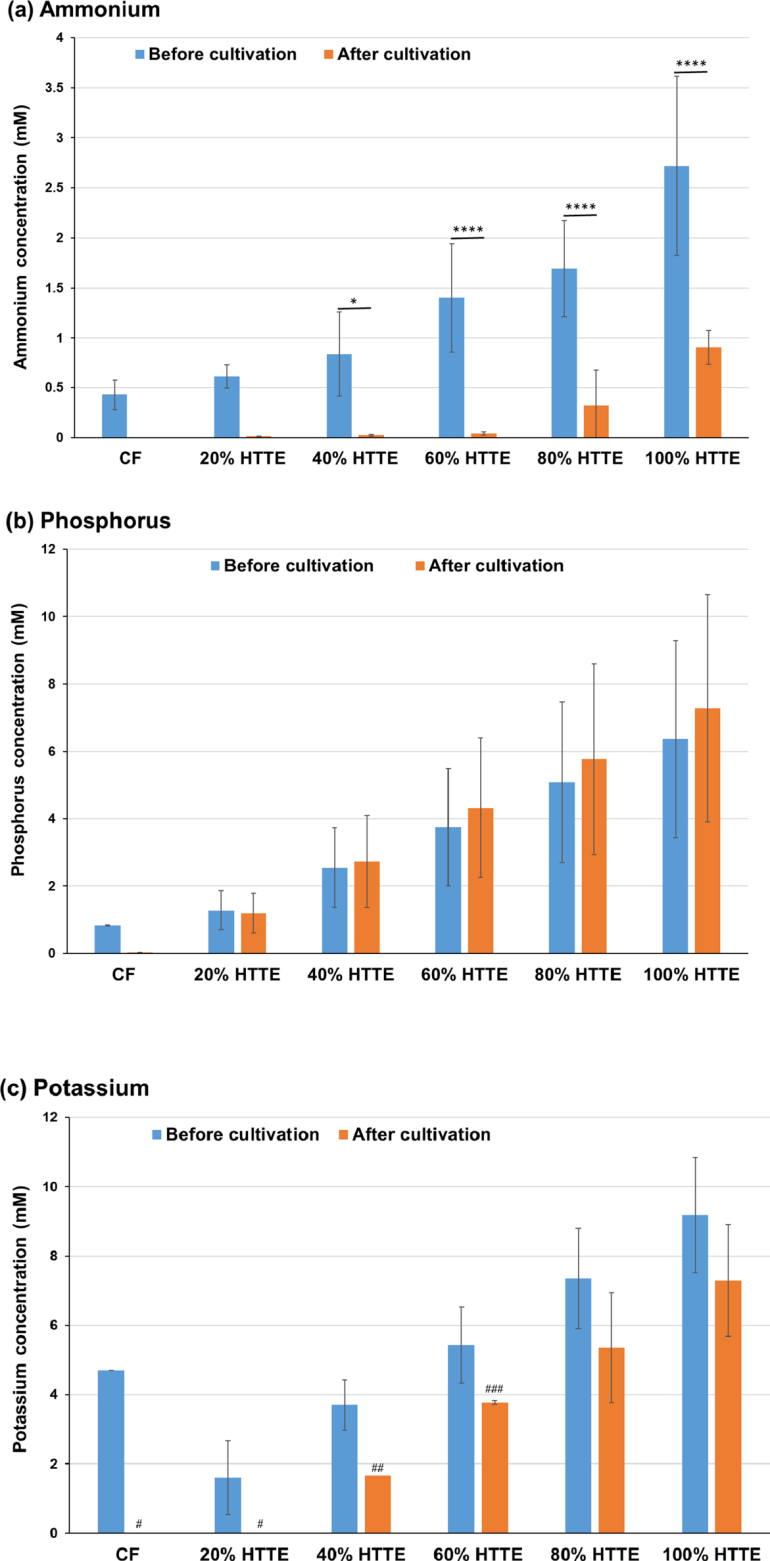

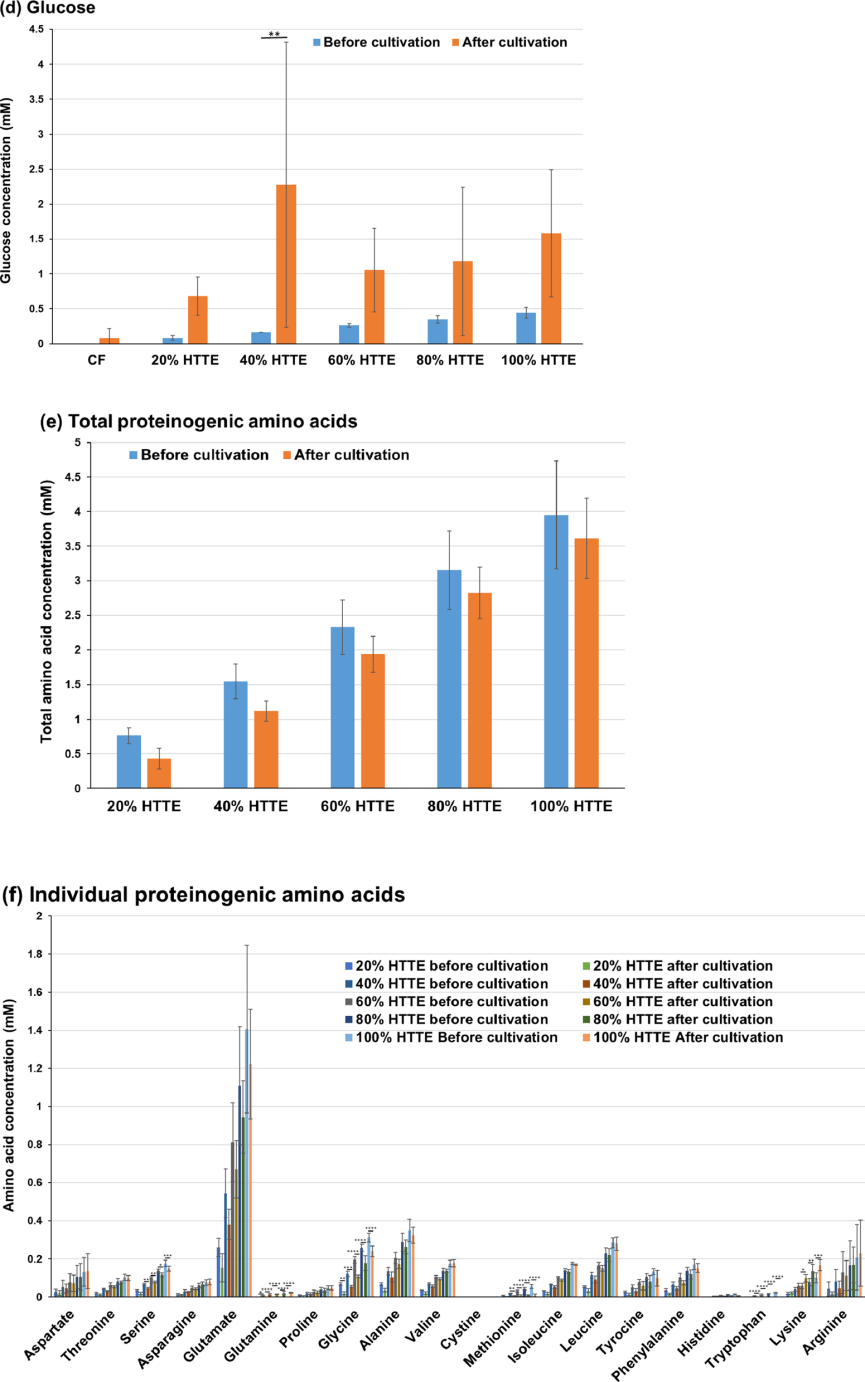
Koshihikari metabolites of ammonium (**a**), phosphorus (**b**), potassium (**c**), glucose (**d**), and proteinogenic amino acids (**e**: total; **f**: individual) measured during cultivation. Data are presented as the mean ± standard deviation (*n* = 4). Statistical analyses were performed to compare before and after cultivations. ^*^: *p* < 0.05; ^**^: *p* < 0.01; ^***^: *p* < 0.001; ^****^: *p* < 0.0001. ^#^ in the potassium data: Four of four trials were undetectable (< 1.5 mM). ^##^: Two of four trials were undetectable (< 1.5 mM), and the data in the graph show the average of two values that were detected. ^###^: One of four trials was undetectable (< 1.5 mM), and the data in the graph show the average and standard deviation of three values that were detected. CF: chemical fertiliser solution; HTTE: heat-treated *Trichormus* extract.

We then investigated the metabolism of Koshihikari. Koshihikari efficiently consumed ammonium in the extract and chemical fertiliser solution (Fig. 5a). Koshihikari also tended to consume phosphorus in the chemical fertiliser solution (Fig. 5b). In addition, Koshihikari efficiently consumed potassium in the extract and chemical fertiliser solution (Fig. 5c). Koshihikari did not consume glucose but rather excreted it (Fig. 5d). This was particularly noticeable for the extracts. Further, Koshihikari tended to consume six proteinogenic amino acids (serine, glutamate, glycine, alanine, methionine, and tryptophan) in the extract (Fig. 5e and f), but excreted a small amount of glutamine and lysine. Overall, the consumption trends of Sasanishiki and Koshihikari were similar.

### Broccoli culture using *Trichormus* extracts and metabolic analysis

**Fig. 6:**
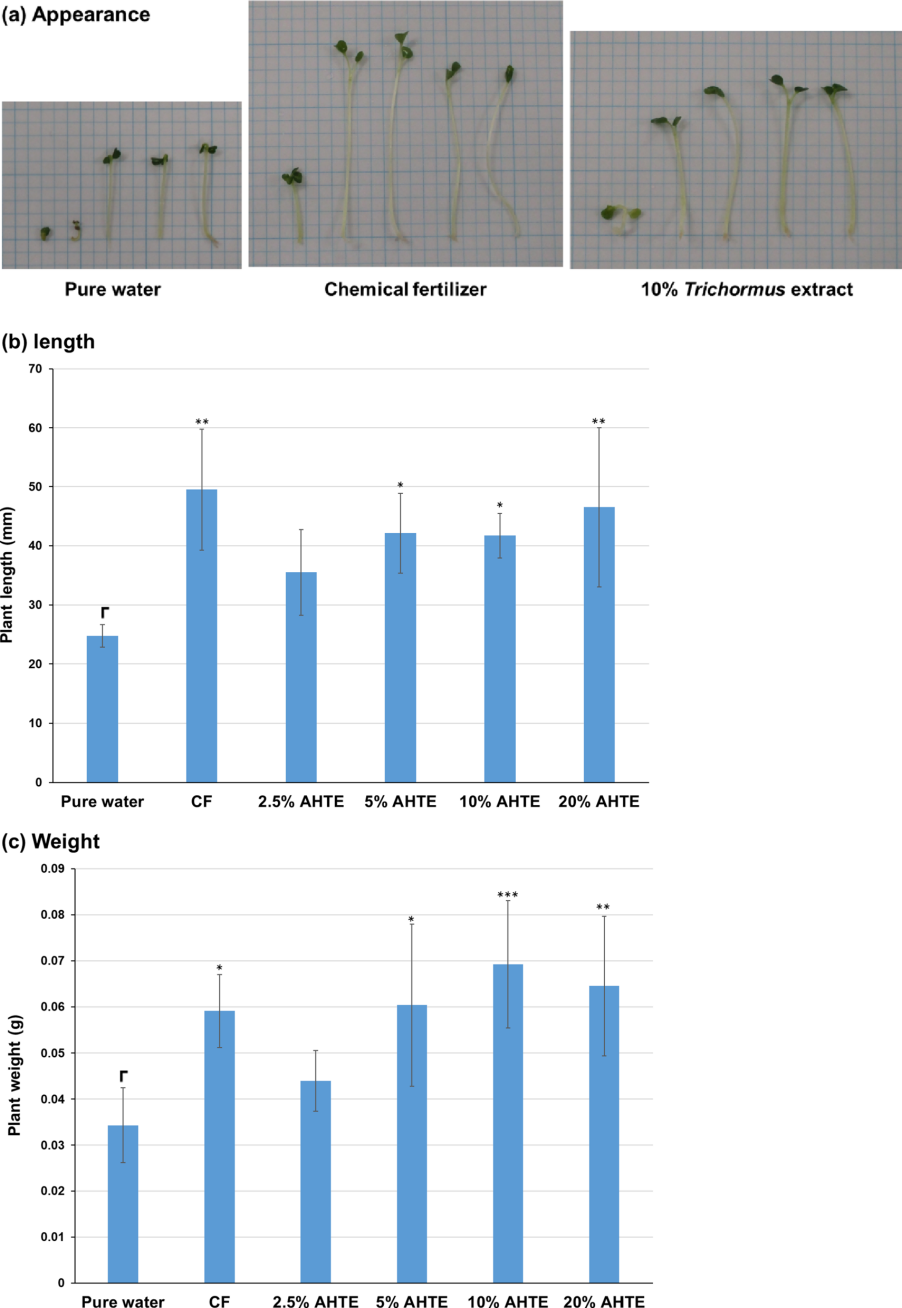
Broccoli cultivation in acid-hydrolysed *Trichormus* extract (2.5–20%). Appearance (**a**), total length (**b**), and total weight (**c**) of broccoli (*Brassica oleracea* var. *italica*) assessed after 14-day-cultivation. Data are presented as the mean ± standard deviation (*n* = 5). Statistical analyses were performed to compare pure-water cultivation with each cultivation condition tested. ^*^: *p* < 0.05; ^**^: *p* < 0.01. CF: chemical fertiliser solution; AHTE: acid-hydrolysed *Trichormus* extract.

Next, we cultured broccoli using the heat-treated *Trichormus* extract. Broccoli sprouts, which can be harvested early after seeding, are attracting attention because they are nutritious and beneficial to human health^6^. Thus, we attempted to harvest them after two weeks of seeding. Of a total of 15 seeds sown in each culture condition tested, 15, 14, 14, 14, 14, and 14 seeds germinated and grew after two weeks in pure water, chemical fertiliser solution, and 20%, 40%, 60%, and 80% extract (heat treatment), respectively. When compared with pure water cultivation, no effects on broccoli length and weight were observed when the heat-treated *Trichormus* extracts (20–80% concentration; *n* = 5) were used (data not shown). Therefore, we cultivated broccoli using the *Trichormus* extract that underwent acid treatment (2.5–20% concentration). Of a total of 25, 25, 26, 25, 26, and 25 seeds (*n* = 5) sown in pure water, chemical fertiliser solution, and 2.5%, 5%, 10%, and 20% extract (acid-hydrolysed), respectively, 25, 24, 24, 25, 25, and 18 seeds germinated and grew after two weeks, respectively; thus, the respective germination rates were 100%, 96%, 92%, 100%, 96%, and 72%, and a decrease in germination rate observed for the 20% extract. The growth of broccoli increased after extract exposure, and the 10–20% extracts displayed 1.7–1.9 and 1.9–2.0 times more length and weight, respectively, compared with those measured during cultivation in pure water (Fig. 6a–c). Broccoli growth with the extracts was comparable with that in the chemical fertiliser group.

**Fig. 7:**
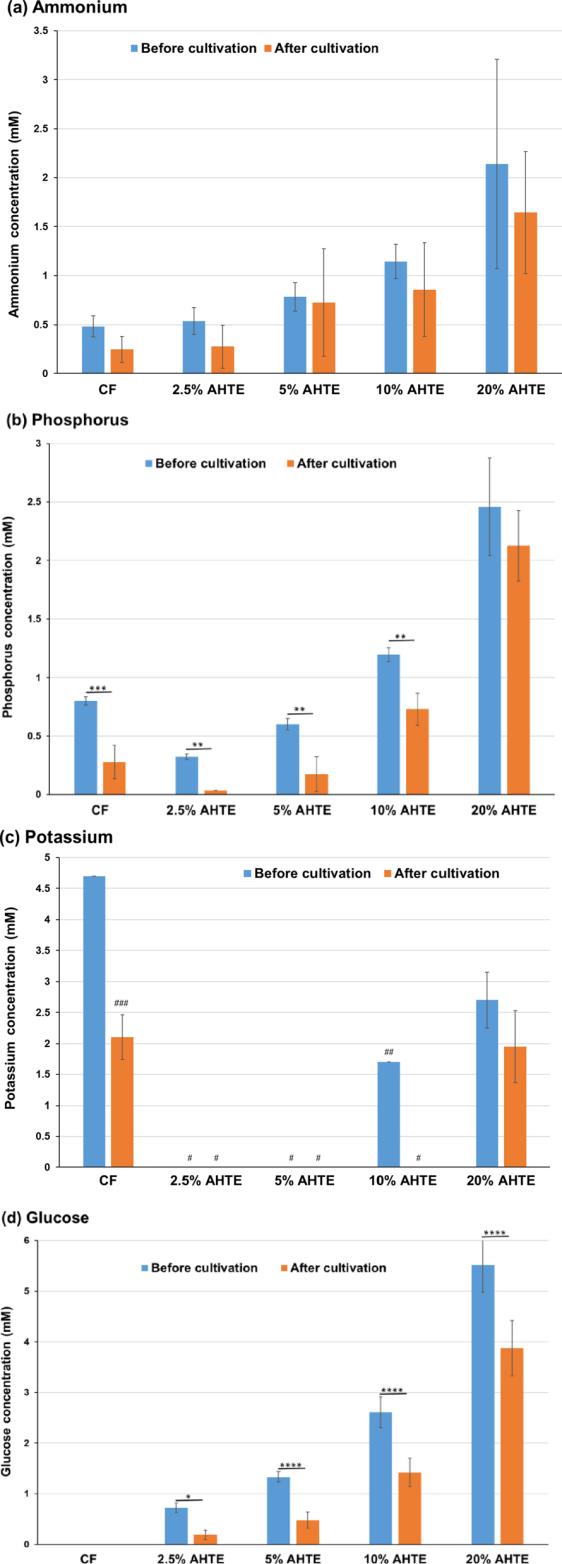

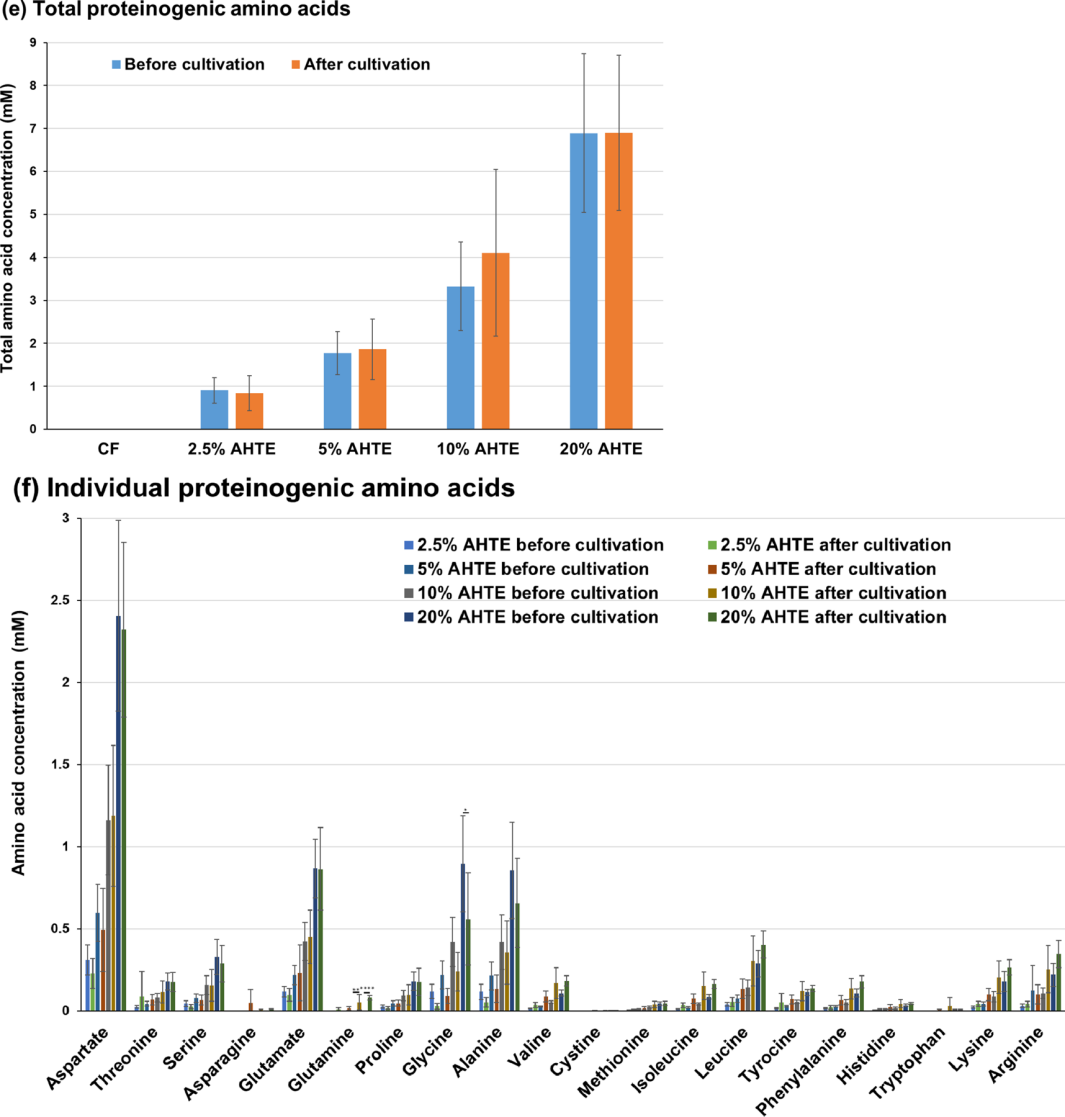
Broccoli metabolites of ammonium (**a**), phosphorus (**b**), potassium (**c**), glucose (**d**), and proteinogenic amino acids (**e**: total; **f**: individual) measured during cultivation. Data are presented as the mean ± standard deviation (*n* = 5). Statistical analyses were performed to compare before and after cultivations. ^*^: *p* < 0.05; ^**^: *p* < 0.01; ^****^: *p* < 0.0001. ^#^ in the potassium data: Five of five trials were undetectable (< 1.5 mM). ^##^: Four of five trials were undetectable (< 1.5 mM), and the data in the graph show one value that was detected. ^###^: One of five trials was undetectable (< 1.5 mM), and the data in the graph show the average and standard deviation of four values that were detected. CF: chemical fertiliser solution; AHTE: acid-hydrolysed *Trichormus* extract.

Next, we investigated broccoli metabolism. Broccoli tended to consume ammonium, phosphorus, and potassium in the extract and chemical fertiliser solution (Fig. 7a–c). It also actively consumed glucose (0.5–1.7 mM; Fig. 7d), showing an intriguing metabolic characteristic unlike rice metabolism at the early growth phase. Regarding amino acids, broccoli tended to consume glycine and alanine and excreted a small amount of glutamine, consistent with rice metabolism.

### Melon culture using *Trichormus* extracts and metabolism analysis

**Fig. 8:**
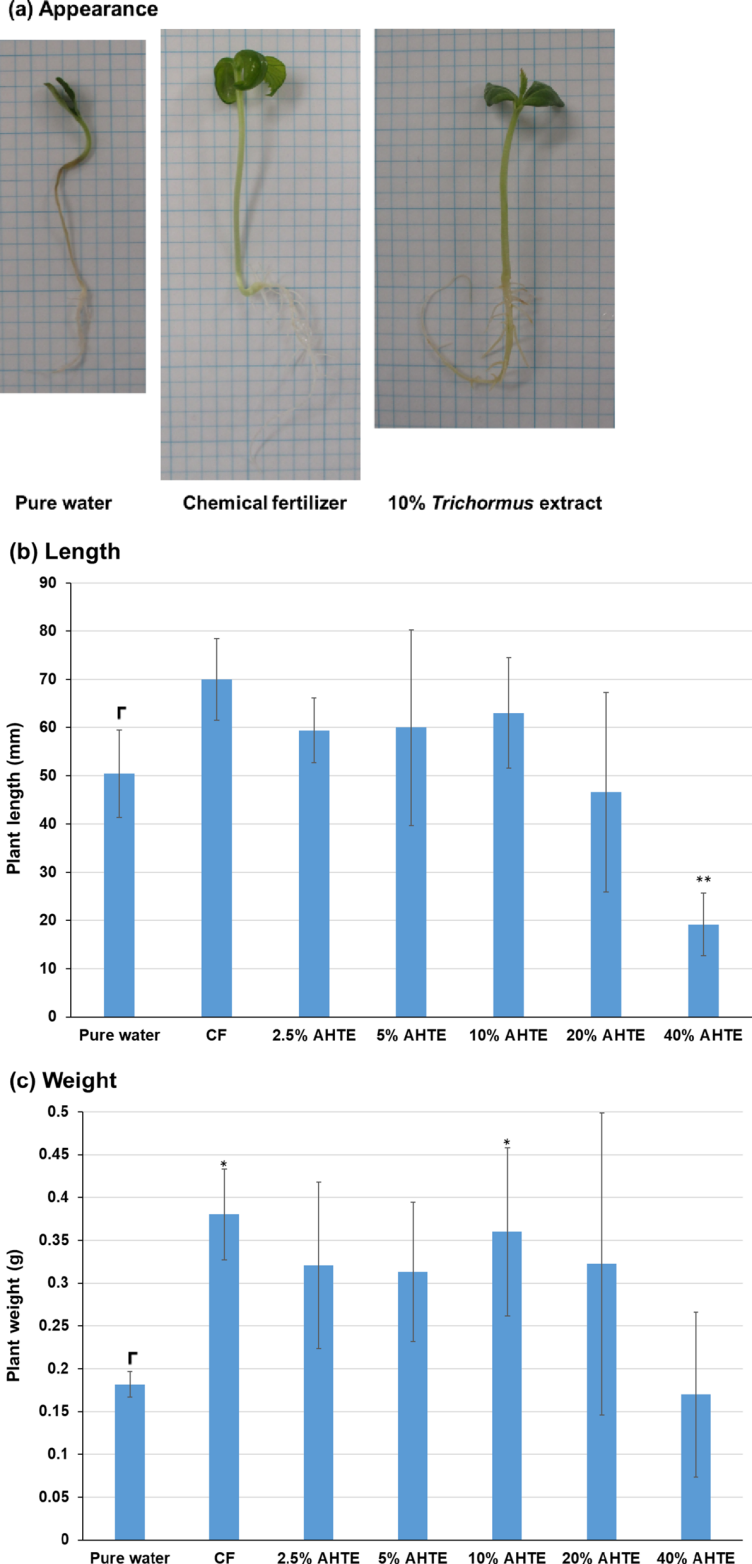
Melon cultivation in acid-hydrolysed *Trichormus* extract (2.5–40%). Appearance (**a**), total length (**b**), and total weight (**c**) of melon (*Cucumis melo* L.) assessed after 21-day-cultivation. Data are presented as the mean ± standard deviation (*n* = 5). Statistical analyses were performed to compare pure-water cultivation with each cultivation condition tested. ^*^: *p* < 0.05; ^**^: *p* < 0.01. CF: chemical fertiliser solution; AHTE: acid-hydrolysed *Trichormus* extract.

Next, we cultured melon using the heat-treated *Trichormus* extract. Compared with pure-water cultivation, the heat-treated *Trichormus* extract (20–100% concentration; *n* = 5) increased melon length by 1.5 times (60% extract) and weight by 1.4 times (80% extract) and had a growth-promoting effect (data not shown). Next, we cultivated melon using the *Trichormus* extract with acid treatment (2.5–40% concentration). The growth of melon increased after extract exposure, and the 10% extracts displayed 1.3 and 2.0 times more length and weight, respectively, compared with those measured during pure-water cultivation (Fig. 8). In the case of melons, the extract was shown to be more effective in increasing plant weight than length. Melon growth with the extracts was also comparable with that in the chemical fertiliser group. However, the 40% extract negatively impacted growth.

**Fig. 9:**
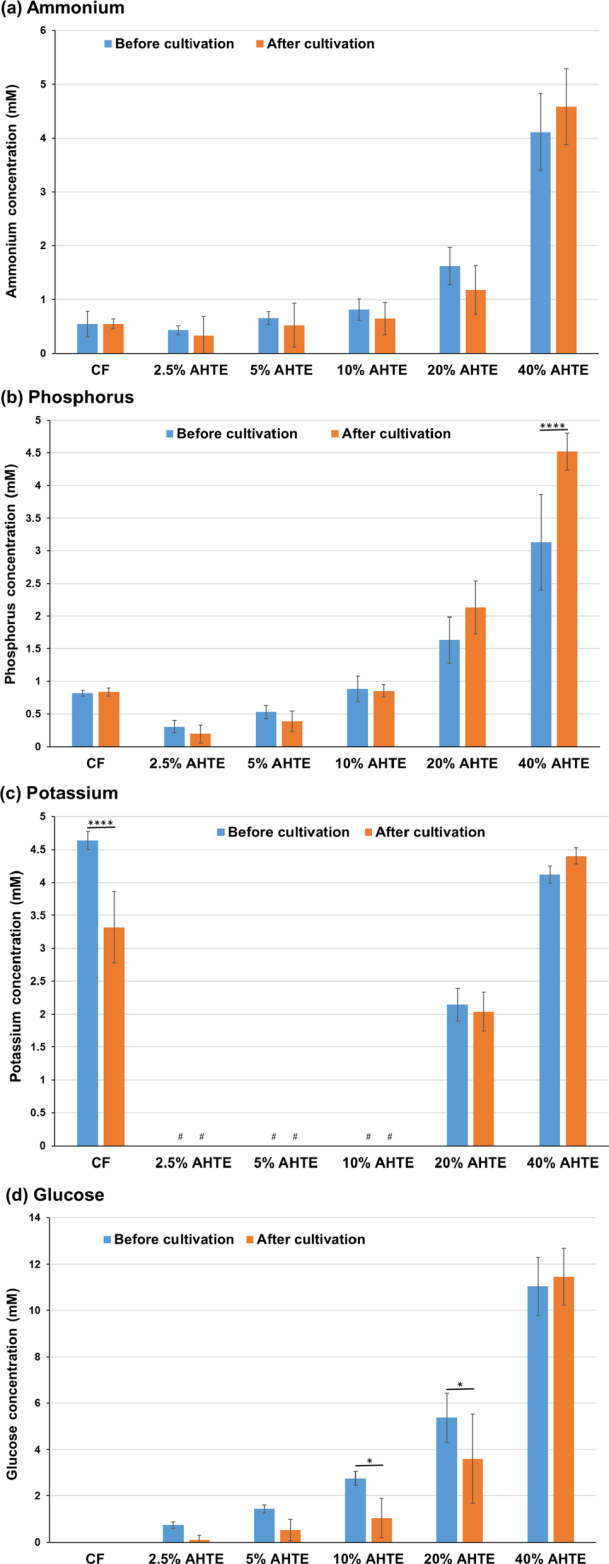

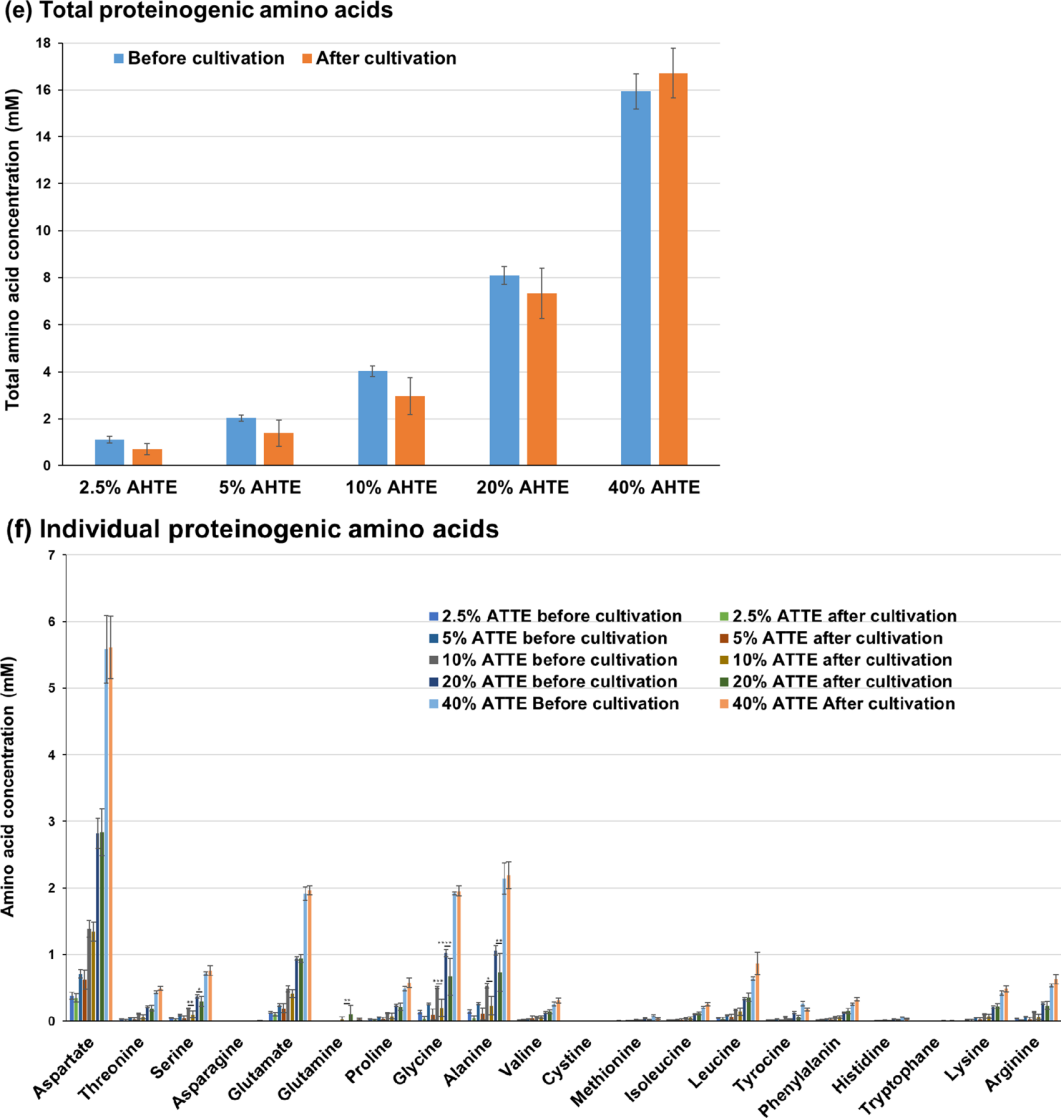
Melon metabolites of ammonium (**a**), phosphorus (**b**), potassium (**c**), glucose (**d**), and proteinogenic amino acids (**e**: total;** f**: individual) measured during cultivation. Data are presented as the mean ± standard deviation (*n* = 5). Statistical analyses were performed to compare before and after cultivations. ^*^: *p* < 0.05; ^****^: *p* < 0.0001. ^#^ in the potassium data: Five of the five trials were undetectable (< 1.5 mM). CF: chemical fertiliser solution; AHTE: acid-hydrolysed *Trichormus* extract.

Finally, we investigated melon metabolism. Melon scarcely consumed ammonium, phosphorus, and potassium (Fig. 9a–c). In contrast, melon consumed glucose (Fig. 9d), serine, glycine, and alanine (Fig. 9f) in the 2.5–10% extracts but not in the 20% extract. This was consistent with its growth rate. However, melon excreted a small amount of glutamine.

## Discussion

Currently, many crops are cultivated in large quantities for food and forage use, including 1,870 million tons of sugarcane, 1,162 million metric tons of corn, 761 million metric tons of wheat, 757 million metric tons of rice, and 353 million metric tons of soybeans^4^ Furthermore, global population growth is predicted to increase food demand by 35–56% between 2010 and 2050^7^. Plants are not only used as food; they are also commercially valuable production systems for (i) biofuel, (ii) pharmaceuticals, and (iii) non-pharmaceuticals, such as antibodies, enzymes, and growth factors for use in research, diagnosis, cosmetic ingredients, biosensors, and biocatalysts^8,9^. The Food and Agriculture Organization of the United Nations (FAO) estimates an additional 50% increase in nitrogen fertiliser demand by 2050^10^. An increase in food production of 50% with a conventional agricultural system will result in an 80% increase in greenhouse gas emissions^11^. Ammonia synthesis via the Haber–Bosch process uses 29–47 MJ of energy and emits 1.5–3 kg CO_2_ per 1 kg of production^12^. The Japanese government has decided to reduce the use of chemical fertilisers by 30% by 2050 (Green Food System Strategy) to achieve carbon neutrality. Additionally, the chemical fertiliser price is currently at a historically high level^13^. In this study, to develop sustainable biological fertilisers, we hydroponically cultured two varieties of rice (Sasanishiki and Koshihikari), which are major cultivars in Japan, broccoli, whose consumption has been increasing worldwide owing to its nutritional benefits^14^, and melon, which is a significant crop that is cultivated worldwide for its juicy fruit^5^, using extracts from the nitrogen-fixing cyanobacterium, *Trichormus* sp. PCC7120. Efficient early growth of these crops was observed when using the extracts (Figs. 2, 4 and 6, and 8, and Supplementary Fig. 2). This report showed that not only the ammonium, phosphorus, and potassium contained in nitrogen-fixing cyanobacterial extracts, but also the glucose and amino acids, contributed to the growth of crops, and that these extracts may thus be useful as fertilisers for crop cultivation. Interestingly, a recent report suggested that an extract of the marine purple photosynthetic bacterium, *Rhodovulum sulfidophilum*, which has CO_2_- and nitrogen-fixing abilities, can be used as a nitrogen source for culturing Japanese mustard spinach^15^. Their method serves as an excellent report that could revolutionise agriculture in the future by making it possible to grow crops without the need for nitrogen fertilisers. In our study, *Trichormus* sp. PCC7120 was cultivated in a medium without a nitrogen source (modified BG11_0_ medium; described in the Methods section) and the extract used to cultivate crops, including two types of rice, which is the fourth most produced crop in the world.

In this study, we used two types of *Trichormus* extracts: heat-treated (100℃, 24 h) and acid-hydrolysed (0.5 N HCl, 100℃, 24 h). High concentrations of acid-hydrolysed extracts cannot be used from the perspective of osmolality (100% heat-treated extract: 52.8 ± 9.6 mOsm/KgH_2_O; 10% acid-hydrolysed extract: 105.0 ± 5.8 mOsm/KgH_2_O) (Supplementary Fig. 1) because high salinity water negatively affected crop growth^16^. In both extracts, ammonium, phosphorus, and potassium, which are important factors for plant cultivation^17^, were present (Fig. 3 and Supplementary Fig. 3). Additionally, glucose and proteinogenic amino acids were present in the *Trichormus* extracts (Fig. 3 and Supplementary Fig. 3). These results showed that the heat-treated extract could effectively supply ammonium, phosphorus, and potassium. In contrast, the acid-hydrolysed extract was efficient at supplying glucose. Sasanishiki and Koshihikari consumed ammonium, potassium, and various amino acids, including aspartate, serine, glutamate, glycine, alanine, and methionine. However, they did not consume glucose (Figs. 3 and 5, and Supplementary Fig. 3). In Sasanishiki cultivation, growth tended to be faster when exposed to the heat-treated extract than when exposed to the acid-hydrolysed extract (Fig. 2 and Supplementary Fig. 2). High growth can likely be obtained by selecting the extract to be used based on the characteristics of the crop. As shown in Figs. 3 and 5, the glucose concentration increased after cultivation (three weeks) compared with the cultivation extracts before rice cultivation, suggesting excess glucose from the rice roots. Some crops have been reported to exude nutrients, including glucose, from their roots^18^. Upland rice secretes glucose from its roots 1–2 weeks after sowing and then later reabsorbs it^19^. These metabolic changes may be linked to efficient rice growth. Using different extracts for different type of crops can potentially accelerate their growth processes. For example, for supplying ammonia, phosphorus, and potassium, which are required from the early stages of growth^17^, the heat-treated extract could be used at the early stages of rice cultivation, and the acid-hydrolysed extract could be used during later stages to supply glucose and inorganic salts.

In contrast, broccoli and melon actively consumed glucose at the early growth phase (Figs. 7 and 9). Plants can incorporate sugars, including glucose, from their roots and reduce the consumption of carbon sources produced during photosynthesis^20^. Broccoli grew faster in acid-hydrolysed extracts than in heat-treated extracts (Fig. 6 and additional data not shown). Additionally, melon tended to grow slightly faster in acid-treated extracts than in heat-treated extracts (Fig. 8 and additional data not shown). When treated with the extract, glucose uptake from roots during the early stages likely accelerated their growth.

In contrast, high salinity water impedes crop growth; salt accumulation inhibits plant growth and reduces the ability to uptake water and nutrients via roots, leading to osmotic or water-deficit stress^16^. In addition, salts cause injury to young leaves and accelerate their senescence because sodium is toxic when accumulated in the cell cytosol, resulting in ionic imbalance and toxicity of transpiring leaves. Salt sensitivity depends on the type of plant and plant growth stages. Generally, plants at early growth stages are more sensitive to salt stress than those at later stages^21^. Salt stress reduces seed germination up to over 50%, either by inhibiting water uptake and/or through the toxic effect of ions^22^. In the cultivation of rice, growth-promoting effects were greatest with the 2.5% extract and somewhat reduced at higher concentrations. Additionally, in the cultivation of melon, growth-promoting effects were greatest with the 10% extract, whereas using the 40% extract negatively impacted growth. In broccoli, a decrease in germination rate was observed (pure water: germination rate = 100%; chemical fertiliser: 96%; 2.5% extract: 92%; 5% extract: 100%; 10% extract: 96%; and 20% extract: 72%). Therefore, when growth was evaluated based on the average value in germinated plants, broccoli growth when using the 10–20% extract showed the highest increase (Fig. 6b and c); however, in averages of the total length and weight of plants in each trial, the 5–10% extract showed the fastest growth (data not shown). More efficient cultivation is possible through improvements made during cultivation. For example, if the seeds are cultivated in pure water until they germinate and then grown in a relatively high salt extract containing high glucose, a higher growth effect might be obtained.

Proteinogenic amino acids, including glutamate, glycine, alanine, and tryptophan, are nitrogen sources that can induce the growth of some crops^23–26^. This study suggests that rice, broccoli, and melon consumed various proteinogenic amino acids (aspartate, serine, glutamate, glycine, alanine, methionine, and tryptophan) (Figs. 3, 5 and 7, and 9, and Supplementary Fig. 3). Proteinogenic amino acids in the *Trichormus* extract may contribute to the growth of some crops.

Cyanobacteria, including *Trichormus*, have been reported to produce plant growth regulators, such as auxin and cytokinin^27–30^. These cyanobacteria-derived factors could promote plant growth, increase root development, and improve plant stress tolerance; thus, this property could be utilised in agriculture. The cyanobacterial extract used in this study may contain plant growth hormone(s) and nutrients, such as ammonia, amino acids, and glucose, where the hormone(s) may have contributed to crop growth.

In contrast to organic manure, chemical fertilisers without carbon sources (Fig. 3d and e) can result in adverse soil and agronomic effects and biogeochemical stoichiometric imbalances^31^. Organic carbon plays important roles in (i) soil structural conservation, fertility, and biological health; (ii) water retention and availability; (iii) reducing the uptake of contaminants, such as cadmium, by crops; and (iv) neutralising the pH in acidic soils^30^. Microalgal extracts contained organic compounds, such as glucose and amino acids, and inorganic compounds, such as ammonium (Figs. 3, 5 and 7, and 9, and Supplementary Fig. 3). Thus, using microalgae-derived extracts has the potential to contribute not only to short-term crop growth but also to long-term farmland fertilisation and biogeochemical stoichiometric balances.

We succeeded in cultivating Sasanishiki, Koshihikari, broccoli, and melon without the need of nitrogen fertilisers by using N_2_ in the air with the help of nitrogen-fixing cyanobacteria; however, phosphorus fertilisers were required for *Trichormus* culture. As phosphorus is a scarce resource that is rapidly declining in nature^32^, this issue must be addressed to ensure the sustainability of our proposed system. Multiple microalgae can grow in wastewater from households or factories, and some are 100% efficient in recovering phosphorus^33^. Thus, it is possible to culture crops with *Trichormus* extracts grown using phosphorus recycled from wastewater to resolve this issue.

In this study, we observed the early cultivation of two leading Japanese rice cultivars, one vegetable, and one type of fruit. Broccoli is harvested for food, particularly as sprouts after 1–2 weeks of cultivation. They were successfully harvested using the *Trichormus* extract in this study (Fig. 6); however, rice and melon require several months to harvest. It would be interesting to challenge the long-term cultivation and harvesting of rice and melon using the *Trichormus* extract and the growth of other crops. Our preliminary experiments aimed at harvesting sugar (sucrose) showed that sugarcane (*Saccharum sinense*) could be cultured in soil for over four months using *Trichormus* sp. (unpublished observation). These challenges will ultimately lead to the development of an effective crop cultivation strategy that eliminates the need for nitrogen fertilisers.

Regarding the scale-up of nitrogen-fixing cyanobacteria culture, Matassa et al. estimated that nitrogen-fixing cyanobacteria grown in open raceway ponds produce approximately 40 metric tonnes (dry weight)/ha/year using only sunlight as the power source^34^. Notably, 1 ha of the cyanobacterial culture could supply nitrogen fertiliser to 100 ha of surrounding agricultural land. Although the cost of this agricultural system is high, it remains appealing for the development of a sustainable farming method without the need for nitrogen fertilisers.

Climate change can negatively affect the crop cultivation, leading to food insecurity^35^. Nitrogen-fixing cyanobacteria hold promise in promoting sustainable agricultural practices^36,37^. This report demonstrated that Sasanishiki, Koshihikari, broccoli, and melon can grow efficiently when using N_2_ and CO_2_ in air with the help of nitrogen-fixing cyanobacteria in the absence of nitrogen fertilisers. Moreover, not only ammonium, phosphorus, and potassium were contained in the nitrogen-fixing cyanobacterial extracts, but also glucose and amino acids, which contributed to the growth of crops. Overall, these extracts may be valuable as fertilisers for crop cultivation.

## Methods

### Cyanobacterial culture and preparation of the *Trichormus* extract

The nitrogen-fixing cyanobacterium, *Trichormus* sp. PCC7120, which was obtained from the Pasteur Cultures of Cyanobacteria (Pasteur Institute, Paris, France), was cultured in an Erlenmeyer flask capped with a silicone plug (AsOne, Osaka, Japan) under continuous light in a plant growth chamber (Biotorn; Nippon Medical & Chemical Instruments, Osaka, Japan) (temperature: 25 °C; CO_2_ concentration: 1%; agitation rate: 60 rpm; and photosynthetic photon flux density [PPFD]: approximately 80 µmol/m^2^/s) using modified BG11_0_ medium, which was completely nitrogen-free (25 mM NaHCO_3_, 620 µM Mg_2_SO_4_, 250 µM CaCl_2_·2H_2_O, 190 µM Na_2_CO_3_, 180 µM K_2_HPO_4_·3H_2_O, 46 µM H_3_BO_3_, 31 µM citric acid, 9.2 µM MnCl_2_·4H_2_O, 6.1 µM FeCl_3_, 2.3 µM Na_2_EDTA-Mg, 1.6 µM Na_2_MoO_4_·2H_2_O, 0.77 µM ZnCl_2_·7H_2_O, 0.32 µM CuSO_4_·5H_2_O, and 0.17 µM CoCl_2_·6H_2_O).

The cyanobacteria were harvested via centrifugation (12,500 × *g*, 5 min), and the supernatant discarded thereafter. Subsequently, medium components were removed via centrifugation (12,500 × *g*, 5 min) twice using pure water (Fujifilm Wako Pure Chemical, Osaka, Japan). Thereafter, the harvested cyanobacteria were lyophilised using a freeze dryer (FDM-1000; Tokyo Rikakikai, Tokyo, Japan), and the *Trichormus* sp. PCC7120 extract prepared using the (i) heat treatment or (ii) acidic hydrolysis methods described below^38^.

(i) Heat treatment method: Lyophilised cyanobacteria were suspended in pure water at a concentration of 50 g/L and heat-treated (100 °C) for 0, 10, or 30 min or 1, 6, or 24 h. After centrifugation (12,500 × *g*, 5 min), the supernatant was used as the *Trichormus* extract of the heat treatment method for crop culture and biochemical analyses.
(ii) Acidic hydrolysis method: Lyophilised cyanobacteria were treated with 0.5 N HCl (Fujifilm Wako Pure Chemical) at a concentration of 50 g/L at 100 °C for 24 h. After neutralisation to pH 6.1 using sodium hydroxide and centrifugation (12,500 × *g*, 5 min), the supernatant was used as the *Trichormus* extract of the acidic hydrolysis method for crop culture and biochemical analyses.

Rice grows in slight-to-weakly acidic (pH 5.5–6.5) conditions, whereas broccoli and melon grow in slightly acidic (pH 6.0–6.5) conditions (data of the Japanese Ministry of Agriculture, Forestry and Fisheries); therefore, the pH of the acid-hydrolysed extracts was adjusted accordingly (pH = 6.1). However, the heat-treated extracts (100%) were slightly acidic (pH = 6.4 ± 0.1; *n* = 13); thus, their pH was not adjusted. The pH of the extracts was analysed using pH test paper (AsOne) and a pH meter (LAQUAtwin; Horiba, Kyoto, Japan).

Regarding the heat-treated extracts, to determine the optimal extraction conditions for *Trichormus*, lyophilised *Trichormus* sp. was heated at 100℃ for 0, 10, or 30 min or 1, 6, or 24 h. Subsequently, we analysed the amounts of ammonium, phosphorus, and potassium present, which are important for crop cultivation. Although the amount of potassium increased after 10 min of heat treatment and remained nearly constant thereafter, the amounts of ammonium and phosphorus increased over time owing to heat treatment and were the highest after 24 h (data not shown). Therefore, we selected a heat-treated extract for 24 h to cultivate the crops. Regarding the acid-hydrolysed extract, after lyophilised *Trichormus* sp. was treated with 0.1 N, 0.25 N, 0.5 N, 1 N, or 2 N HCl at 100 °C for 24 h, the nutrients in the extracts were analysed. Although the total amino acid concentration increased depending on the acid concentration, the glucose concentration was highest at 0.5–1 N (data not shown); therefore, considering the osmolality, a 0.5 N HCl-treated extract was used. Regarding osmolality, the osmolalities of the 100% heat-treated and 5% acid-hydrolysed (0.5 N HCl-treated) extracts were approximately equal (100% heat-treated extract: 52.8 ± 9.6 mOsm/KgH_2_O; 5% acid-hydrolysed extract: 52.0 ± 2.2 mOsm/KgH_2_O) (Supplementary Fig. 1); thus, a 2.5–20/40% extract was used because a highly concentrated acid-hydrolysed extract may have adverse effects on crop growth owing to its high osmolality.

### Crop culture

The seeds of two types of rice (*O. sativa* L. ‘Sasanishiki’ and ‘Koshihikari’), which are leading Japanese cultivars^39,40^, were purchased from Nohken (Kyoto, Japan), and those of broccoli (for broccoli sprouts) purchased from Sakataseed (Kanagawa, Japan). In the case of melon, nursery plants (Korotan, Sakataseed) were purchased, and the seeds used after harvesting. Sasanishiki and Koshihikari were precultured in 60 mm culture dishes (Corning, Corning, NY, USA). After 4–5 days, germination was observed, and plants of similar lengths randomly selected. The two plants were sown in a sponge for hydroponics (Trusco, Tokyo, Japan) soaked (i) in pure water, (ii) with chemical fertiliser (HYPONeX Japan, Osaka, Japan), or (iii) with the *Trichormus* extract in a cultivation tube with a cap (AGC Techno Glass, Shizuoka, Japan) for plant cultivation. The ratio of ammonium nitrogen to nitrate nitrogen was 2.90:1.05 in the chemical fertiliser (HYPONeX Japan data); therefore, most of the nitrogen source available was in the form of ammonium. The crops were cultured using fluorescent lights for growing plants (NEC, Tokyo, Japan), with a light: dark period of 16 h:8 h (temperature: approximately 25℃; PPFD: approximately 10 µmol/m^2^/s), for 21 days after seeding. Broccoli was grown directly with the main culture, and pre-cultivation not performed. In the experimental group using the heat-treated extract, a total of 15 seeds were sown (*n* = 5), and in the acidic hydrolysis treatment, a total of 25–26 seeds were sown (*n* = 5). The seeds were directly sown into the sponge for hydroponics soaked in each extract group in the tube and cap for plant cultivation (Kluma; Amazon Japan, Tokyo, Japan) and cultured for 14 days under the same conditions as those used for rice. Melon was precultured in 60 mm culture dishes. After 3–5 days, germination was observed, and plants of similar lengths randomly selected. The plants that were sown in the sponge for hydroponics were cultured under the same conditions as those used for rice for 21 days after seeding. After cultivation, plant lengths were measured using a stainless-steel ruler (AsOne) from the stem’s base to the leaf’s tip after straightening all crooked crops. After thoroughly removing moisture adhering to the crop with a paper towel (Konya Paper, Shizuoka, Japan), the weights were measured using an electronic balance (ME614S; Sartorius, Göttingen, Germany). The cultivation extracts before and after cultivation were centrifuged (12,500 × *g*, 5 min), and the supernatant subjected to biochemical analysis. The lengths and weights of the plants were calculated as the average of the lengths and weights of the germinated/growth seeds in each trial.

#### Biochemical analyses

Biochemical analyses were performed, as described in previous studies^38,41–43^. Ammonium, amino acid, glucose, phosphorus, and potassium levels were determined using colorimetry, liquid chromatography-mass spectrometry, hexokinase UV, direct colorimetric determination, and ion electrode methods. Osmolality was determined through cryoscopy. These biochemical analyses were conducted at an outsourced laboratory (SRL, Tokyo, Japan). The detection limits for each factor are as follows: ammonium: 0.041 mM; glucose: 0.056 mM; phosphorus: 0.032 mM; potassium: 1.5 mM.

##### Statistical analysis

Multigroup comparisons were performed using GraphPad Prism software v.10.1.2 (GraphPad Software, Inc., La Jolla, CA, USA). Multiple comparisons were conducted using analysis of variance, followed by Tukey’s or Sidak’s tests, as appropriate. A *p*-value < 0.05 was considered statistically significant.

## Electronic supplementary material

Below is the link to the electronic supplementary material.

Supplementary Material 1

Supplementary Material 2

Supplementary Material 3

Supplementary Material 4

## Data Availability

Data are available from the corresponding authors on reasonable request.
