# Supplementary material for: Crop cultivation without nitrogen fertiliser using nitrogen-fixing cyanobacterial extracts for low environmental impact
Source: Sci Rep. 2025 May 26;15:18365. doi: 10.1038/s41598-025-01741-5 (PMC12106771; doi:10.1038/s41598-025-01741-5)
Supplement: Supplementary file 1 — Supplementary Material 1 [file 41598_2025_1741_MOESM1_ESM.pdf]

## Supplementary Table 1 | Experimental procedure used for each figure

| Figure No. | *Trichormus* extract (concentration) | Crop name | Analysis items |
|---|---|---|---|
| Figure 2 | Heat treatment method (20, 40, 60, 80, 100%) | *Oryza sativa* L. 'Sasanishiki' (rice) | Growth |
| Figure 3 | Heat treatment method (20, 40, 60, 80, 100%) | *Oryza sativa* L. 'Sasanishiki' (rice) | Metabolism |
| Figure 4 | Heat treatment method (20, 40, 60, 80, 100%) | *Oryza sativa* L. 'Koshihikari' (rice) | Growth |
| Figure 5 | Heat treatment method (20, 40, 60, 80, 100%) | *Oryza sativa* L. 'Koshihikari' (rice) | Metabolism |
| Figure 6 | Acid hydrolysis method (2.5, 5, 10, 20%) | *Brassica oleracea* var. *italica* (broccoli) | Growth |
| Figure 7 | Acid hydrolysis method (2.5, 5, 10, 20%) | *Brassica oleracea* var. *italica* (broccoli) | Metabolism |
| Figure 8 | Acid hydrolysis method (2.5, 5, 10, 20, 40%) | *Cucumis melo* L. (melon) | Growth |
| Figure 9 | Acid hydrolysis method (2.5, 5, 10, 20, 40%) | *Cucumis melo* L. (melon) | Metabolism |
| Supplementary Figure 1 | Heat treatment method (20, 40, 60, 80, 100%) Acid hydrolysis method (2.5, 5, 10, 20%) | - | Osmolality |
| Supplementary Figure 2 | Acid hydrolysis method (2.5, 5, 10, 20%) | *Oryza sativa* L. 'Sasanishiki' (rice) | Growth |
| Supplementary Figure 3 | Acid hydrolysis method (2.5, 5, 10, 20%) | *Oryza sativa* L. 'Sasanishiki' (rice) | Metabolism |
