# Supplementary material for: Crop cultivation without nitrogen fertiliser using nitrogen-fixing cyanobacterial extracts for low environmental impact
Source: Sci Rep. 2025 May 26;15:18365. doi: 10.1038/s41598-025-01741-5 (PMC12106771; doi:10.1038/s41598-025-01741-5)
Supplement: Supplementary file 2 — Supplementary Material 2 [file 41598_2025_1741_MOESM2_ESM.pdf]

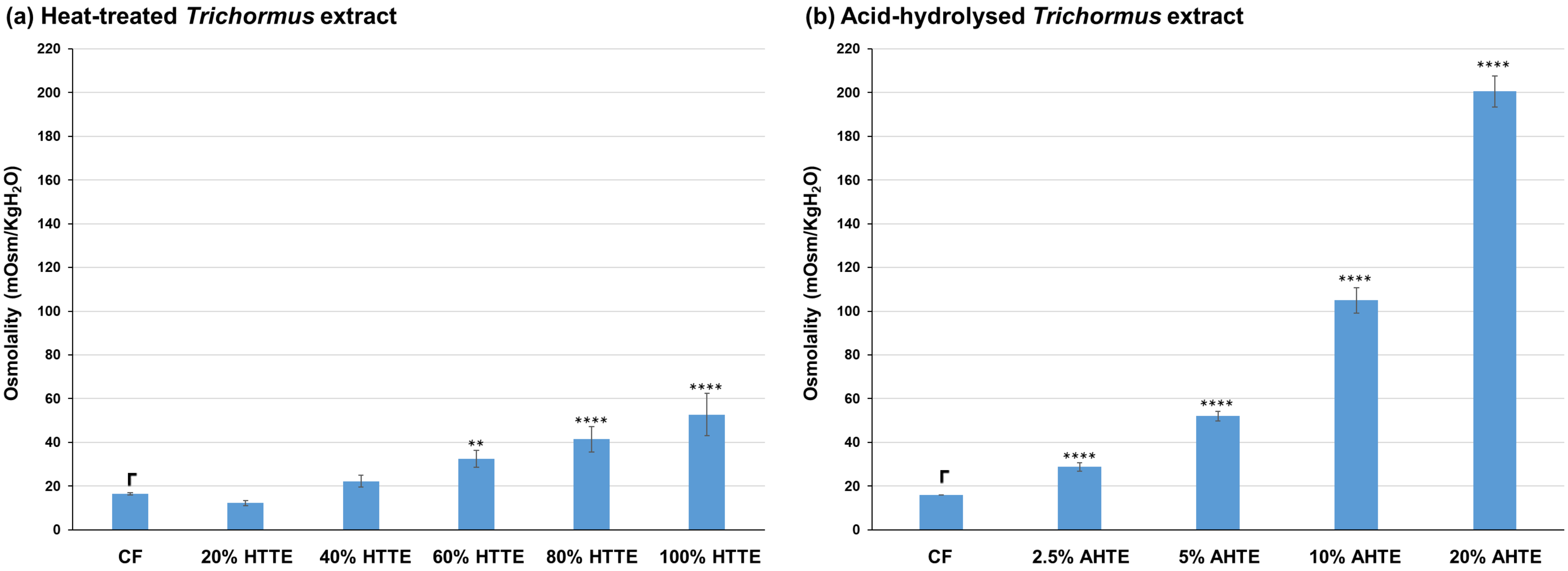

Supplementary Fig. 1 | Osmolality of the heat-treated (a) or acid-hydrolysed (b) *Trichormus* extract. Data are presented as the mean ± standard deviation (n = 4). Statistical analysis was performed to compare the chemical fertiliser solution and each cultivation condition. **: p <0.01; ****: p <0.0001. CF: chemical fertiliser solution; HTTE: heat-treated *Trichormus* extract; AHTE: acid-hydrolysed *Trichormus* extract.
