# Supplementary material for: Crop cultivation without nitrogen fertiliser using nitrogen-fixing cyanobacterial extracts for low environmental impact
Source: Sci Rep. 2025 May 26;15:18365. doi: 10.1038/s41598-025-01741-5 (PMC12106771; doi:10.1038/s41598-025-01741-5)
Supplement: Supplementary file 3 — Supplementary Material 3 [file 41598_2025_1741_MOESM3_ESM.pdf]

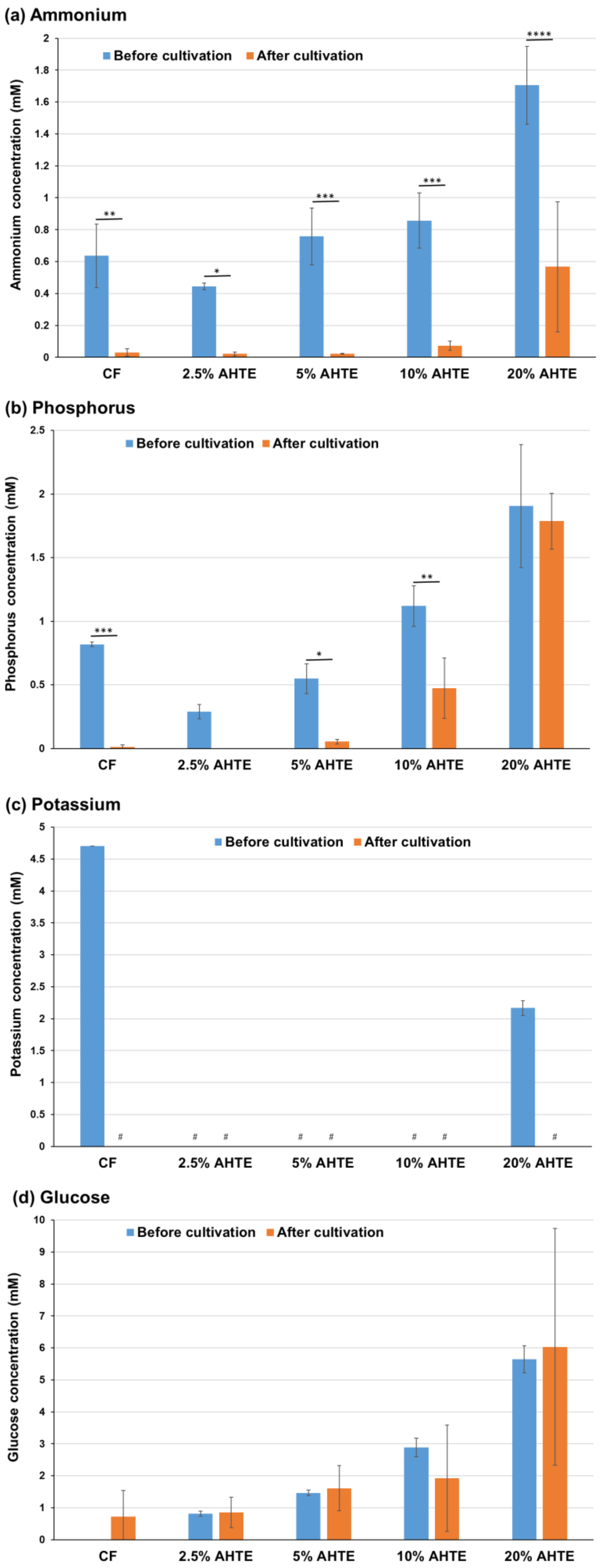
(a) Ammonium
Before cultivation
After cultivation
Ammonium concentration (mM)
2
1.8
1.6
1.4
1.2
1
0.8
0.6
0.4
0.2
0
**
*
***
***
****
CF
2.5% AHTE
5% AHTE
10% AHTE
20% AHTE
(b) Phosphorus
Before cultivation
After cultivation
Phosphorus concentration (mM)
2.5
2
1.5
1
0.5
0
***
*
**
CF
2.5% AHTE
5% AHTE
10% AHTE
20% AHTE
(c) Potassium
Before cultivation
After cultivation
Potassium concentration (mM)
5
4.5
4
3.5
3
2.5
2
1.5
1
0.5
0
CF
2.5% AHTE
5% AHTE
10% AHTE
20% AHTE
(d) Glucose
Before cultivation
After cultivation
Glucose concentration (mM)
10
9
8
7
6
5
4
3
2
1
0
CF
2.5% AHTE
5% AHTE
10% AHTE
20% AHTE

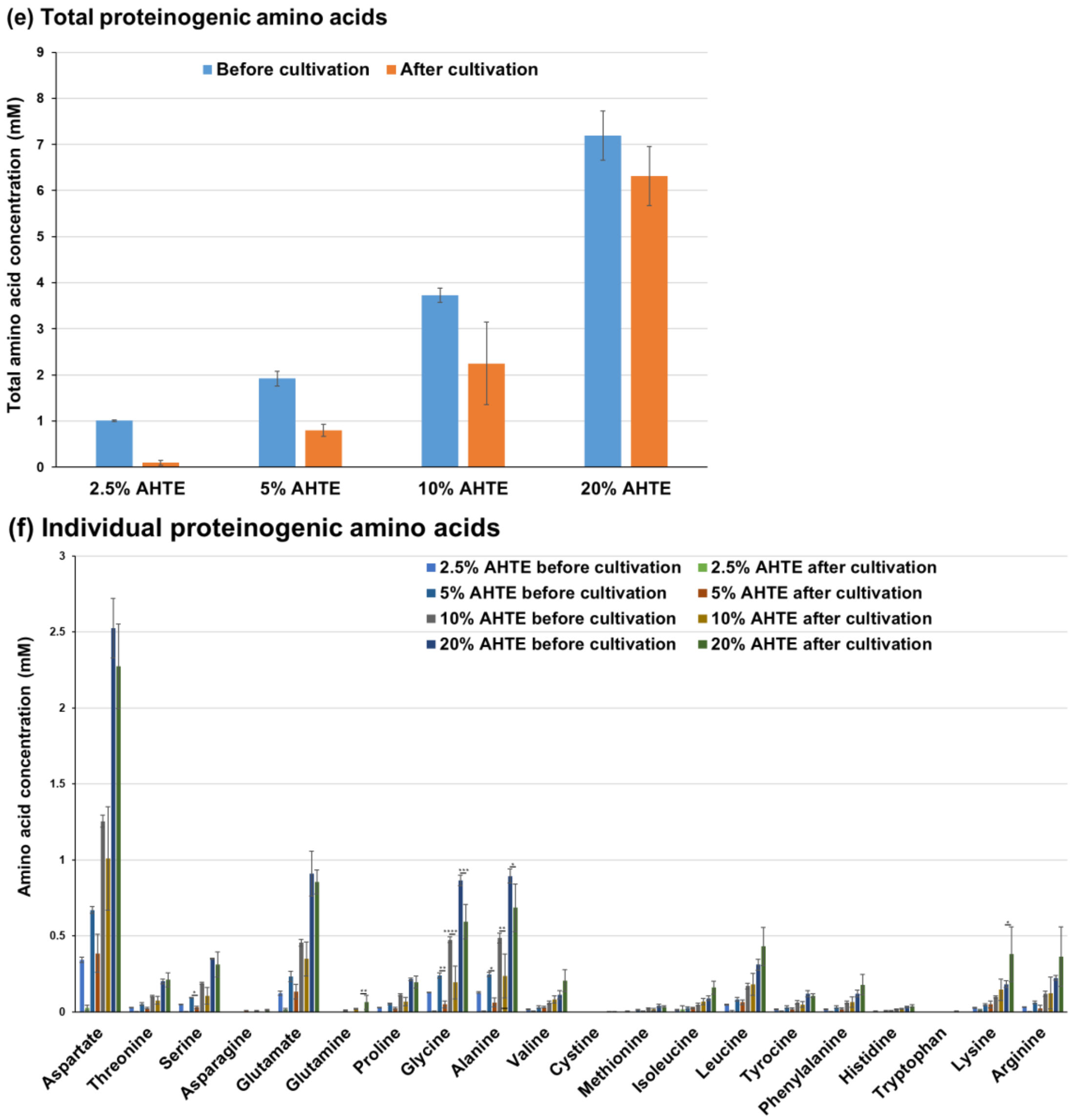

Supplementary Fig. 3 | Sasanishiki metabolites of ammonium (a), phosphorus (b), potassium (c), glucose (d), and proteinogenic amino acids (e: total; f: individual) measured during cultivation (acid-hydrolysed *Trichormus* extract). Data are presented as the mean ± standard deviation (n = 3). Statistical analyses were performed to compare before and after cultivations. *: p <0.05; **: p <0.01; ***: p <0.001; ****: p <0.0001. $^{\#}$ in the potassium data: Three of the three trials were undetectable (<1.5 mM). CF: chemical fertiliser solution; AHTE: acid-hydrolysed *Trichormus* extract.
