# Supplementary material for: Crop cultivation without nitrogen fertiliser using nitrogen-fixing cyanobacterial extracts for low environmental impact
Source: Sci Rep. 2025 May 26;15:18365. doi: 10.1038/s41598-025-01741-5 (PMC12106771; doi:10.1038/s41598-025-01741-5)
Supplement: Supplementary file 4 — Supplementary Material 4 [file 41598_2025_1741_MOESM4_ESM.pdf]

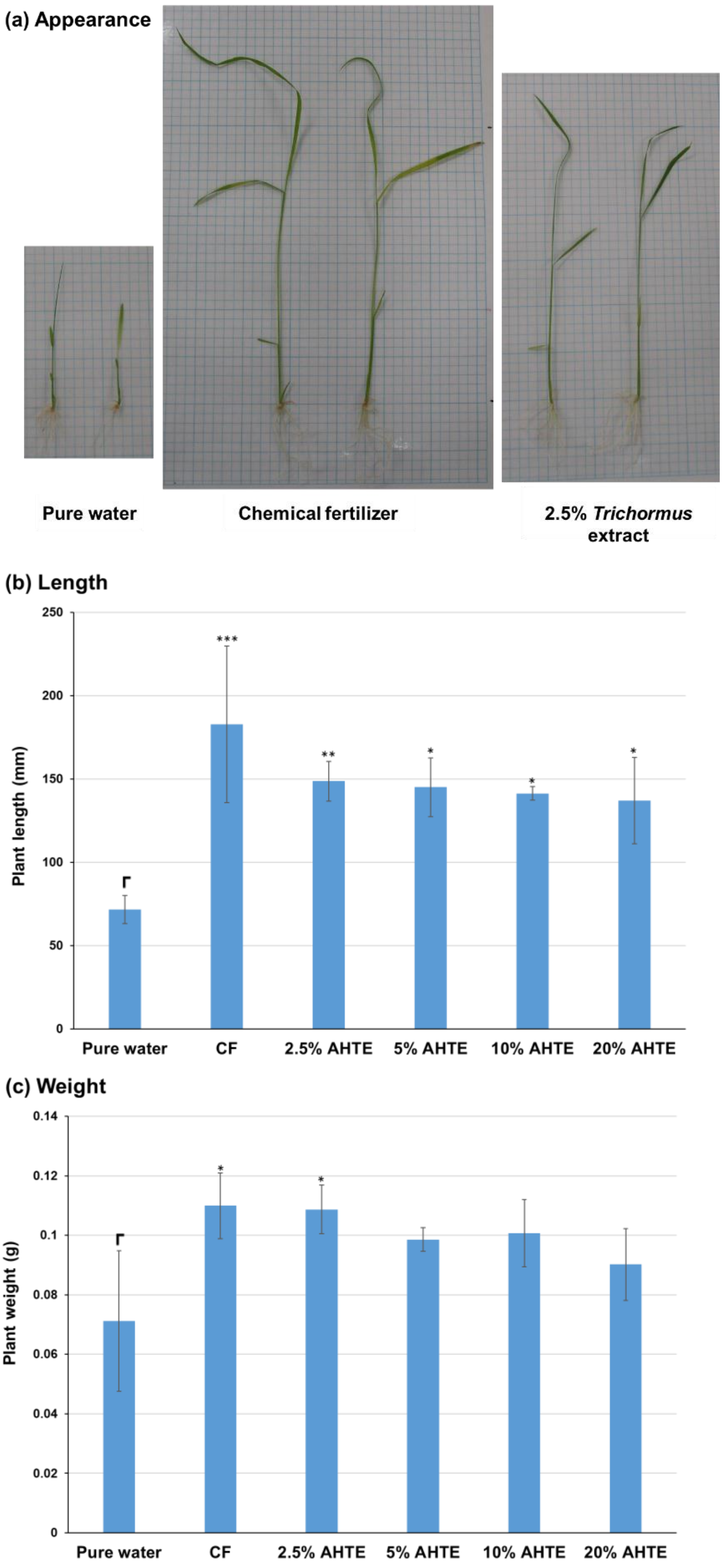

Supplementary Fig. 2 | Sasanishiki cultivation in acid-hydrolysed *Trichormus* extract (2.5–20%). Appearance **(a)**, length **(b)**, and weight **(c)** of *Oryza sativa* L. ‘Sasanishiki’ assessed after 21-day-cultivation. Data are presented as the mean ± standard deviation (n = 3). Statistical analyses were performed to compare pure-water cultivation with each cultivation condition tested. *: p <0.05; **: p <0.01; ***: p <0.001. CF: chemical fertiliser solution; AHTE: acid-hydrolysed *Trichormus* extract.
